# NLRP3-inflammasome activation is associated with epithelial-mesenchymal transition and progression of colorectal cancer

**DOI:** 10.22038/ijbms.2021.52355.11835

**Published:** 2021-04

**Authors:** Yasser Marandi, Shahryar Hashemzade, Heidar Tayebinia, Jamshid Karimi, Alireza Zamani, Iraj Khodadadi

**Affiliations:** 1 Department of Clinical Biochemistry, Faculty of Medicine, Hamadan University of Medical Sciences, Hamadan, Iran; 2 Department of General Surgery, Imam Reza Hospital, Tabriz University of Medical Sciences, Tabriz, Iran; 3 Department of Immunology, Faculty of Medicine, Hamadan University of Medical Sciences, Hamadan, Iran

**Keywords:** Colorectal neoplasms, Epithelial-mesenchymal – transition, Inflammasome, NLRP3, Transforming growth - factor-β

## Abstract

**Objective(s):**

Since activation of NLRP3 inflammasome results in the production of interleukin-1β (IL 1β) and initiation of inflammation as the key players in development of cancer, this study investigated possible activation of NLRP3 inflammasome during the progression of colorectal cancer (CRC) and evaluated the role of NLRP3 inflammasome in epithelial-mesenchymal transition (EMT) process.

**Materials and Methods:**

Tissue samples were collected from cancerous (test) and adjacent normal tissues (control) of forty-three male CRC patients (18 grade I and 25 grade III). The gene expression and protein levels were determined by qRT PCR and Western blotting, respectively, and tissue morphological was examined by histopathology.

**Results:**

The gene and protein expression levels of transforming growth factor-β (TGF β), IL 1β, nuclear factor κB (NF κB), NLRP3, and caspase-1, as well as the enzyme activity of caspase-1, were significantly increased in CRC. mRNA and protein levels of TGF-β, mature IL 1β, NF κB, and NLRP3 were higher in patients with grade III. EMT markers N cadherin, vimentin, and MMP 9 markedly increased in CRC, and were higher in grade III than grade I, whereas expression of E-cadherin declined by the progression of CRC. NLRP3 protein level was inversely correlated with E-cadherin whereas it positively was correlated with IL 1β, active NF κB, N cadherin, vimentin, and MMP 9.

**Conclusion:**

This study for the first time showed that activation of NLRP3 inflammasome contributed to the progression of CRC and is correlated with the EMT process. Although the present study showed that EMT markers are positively correlated with tumor grade, further investigations are required to strongly link the EMT markers to the progression of CRC.

## Introduction

Colorectal cancer (CRC) is the third most common cancer and the second leading cause of cancer-related deaths worldwide, with an estimated 1.8 million new cases diagnosed annually (1). CRC represents a multifactorial disease resulting from a combination of genetic and environmental factors. Its incidence is high in developed countries, and factors such as intestinal disorders, high fat diet, smoking, consumption of red meat and processed meat-rich diets, excessive alcohol consumption, and physical inactivity are predominant contributors to CRC (2, 3). The stage of disease at diagnosis is the main prognostic marker of cancer survival. For example, the five-year survival rate is up to 90% for grade I, but it is less than only 15% for grade IV (4). The genetic alterations can be classified as chromosomal instability, microsatellite instability, and CpG island methylator phenotype (5). The epithelial-mesenchymal transition (EMT) is a process by which epithelial cells lose their polarity and are converted to a more invasive (mesenchymal) phenotype (6). Emerging evidence indicates that the EMT process plays a key role in the progression and metastasisthe colorectal cancer (6) and it is not surprising that inhibition of EMT-associated transcription factors can be an attractive strategy for reducing the risk of cancer progression (7). The results of numerous studies in the last decade have shown that transforming growth factor-beta (TGF-β) and tumor necrosis factor-α (TNF-α) play pivotal roles in the formation of EMT in colorectal cancer (8). TGF-β, a potent inducer of cancer-associated EMT (9), is a cytokine with known involvement in numerous cellular processes, including adhesion, immune regulation, apoptosis, invasion, proliferation, and differentiation (10).

NLRP3 inflammasome is a multiprotein comphichthat induces three types of caspase-1-mediated responses: interleukin-1β (IL-1β), IL-18, and pyroptosis (11). It has been reported that pulmonary inflammation and fibrosis can be attenuated by inhibiting activation of NLRP3-inflammasome (12). The canonical effects of NLRP3 contribute to production of IL-1β and IL-18, but the non-canonical effects of NLRP3 drive cells to the EMT process and fibrosis (13). Additionally, in colon cancer cells overexpression of NLRP3 can directly promote TGF-β and R-Smad activation (14, 15). 

To the best of our knowledge, despite clinical and pre-clinical evidence for the participation of TGF-β in the EMT process, no clinical study has yet addressed the correlation of NLRP3-mediated EMT and the grade of colorectal cancer. Therefore, the present study investigates possible roles of TGF-β, NLRP3, and NLRP3 inflammasome complex in mediating the EMT process by quantitatively determining the genes and/or proteins involved in the activation of TGF-β and NLRP3 pathways and EMT.

## Materials and Methods

***Patients and sample collection***

Fortycutive male subjects aged 42-73 years (means 54±5 years) were recruited into the study at the Imam Reza Hospital (Tabriz University of Medical Sciences, abriz-, Iran) between April 2016 and October 2018. Colorectal cancer was diagnosed by a specialist and histopathological examinatiandthe tumor types and grades were pathologically classified according to the UICC-TNM classification system (16). Among 43 recruited subjects, 36 patients had pr tumortumors located in the colon (21 with gradeIII and 15 with gradeI) and 7 had pr tumortumors located in the rectum (4 with gradeIII and 3 withgrade I). Overall, twenty-five patients (58.2%) were diagnosed as gradeIII and eighteen subjects (41.8%) were classified as of CRC. Patients with any previous diagnosis of inflammatory bowel disease (IBD), type 2 diabetes, cardiovascular disease, and those with a history of radiotherapy or chemotherapy treatment were excluded from the study. 

Cancerous tissues and normal tissues adjacent to the cancer tumors were obtained from all patients at the time of surgery. Tissue samples were gently rinsed in ice-cold phosphate-buffered saline (PBS) to remove excess blood, rapidly frozen in liquid nitrogen, and stored at -80 °C for future analysis. The study was approved by the Research Ethics Committee of Hamadan University of Medical Sciences (Hmadan, Iran, Ethics approval number: IR-UMSHA-REC.1396.916) and all procedures performed in the study were in accordance with the 1964 Declaration of Helsinki (version 2013). The nature of the study was explained to patients and infs wereconsent was obtained from all applicants.

***Determination of gene expression level by qRT-PCR***

To determine gene expression levels of TGFβ, NLRP3, IL1β, NFκB, and caspase1 total RNA was extracted from tissues using the RNeasy Plus Universal Mini Kit (Qiagen, Germantown, MD, USA), according to the manufacturer’s instructions. Briefly, up to 40 mg of frozen tissue was homogenized in the lysis solution. The lysate was filtered and loaded onto the RNA-binding column. The column was washed with washing solutions and RNA was eluted by adding 50 μl of RNase-free water to the membrane of the RNA-binding column. The quantity and quality of extracted RNA were checked using a NanoDrop spectrophotometer (Thermo Fisher Scientific, MAUSA) and 1% TAE-agarose electrophoresis, respectively. TGFβ, NLRP3, IL1β, NFκB, and caspase1 mRNA levels were measured using SYBR Green Real-Time PCR Master Mix (Ampliqon, OdenseDenmark) using gene-specific forward and reverse primer pairs (Table 1). Data were normalized to the expression of GAPDH (as housekeeping gene) and fold changes in gene expression were calculated according to the 2^ΔΔCT^ method. 

***Determination of protein levels by Western blotting***

To assess TGFβ, NLRP3, mature IL1β, pNFκB p65, procaspase1, ASC, vimentin, Ncadherin, Ecadherin, and MMP9, total protein was extracted from the frozen tissue samples using RIPA lysis buffer (Santa Cruz Biotechnology, California-USA), as previously described (17). Briefly, tissues were homogenized for 5 min on ice in lysis buffer containing the protease and phosphatase inhibitors to obtain total protein. After centrifugation at 15,000 rpm for 40 min at 4 °C, the supernatants were collected and stored at -20 °C until assay. Total protein concentration was determined using the Bradford protein assay kit (Bio-Rad, Hercules, California-USA). An equal amount of protein extracts (g) werewas separated by electrophoresis on SDS-PAGE gels and then transferred to polyvinylidene difluoride (PVDF) membranes (Millipore, MAUSA). After blocking with 5% non-fat dried milk in Tris-buffered saline-Tween 20 (TBS-T) bufferfor 1 hhr at room temperature, the membranes were incubated with mouse anti-TGFβ1 antibody (Santa Cruz, sc-130348), mouse anti-IL1β antibody (Santa Cruz, sc-12742), rabbit antiNLRP3 antibody (Abcam, ab214185), mouse antipNFkB p65 antibody (Santa Cruz, sc-136548), mouse antiNcadherin antibody (Santa Cruz, sc-8424), mouse antiEcadherin antibody (Santa Cruz, sc-8426), mouse anti-vimentin antibody (Santa Cruz, sc-373717), rabbit anti-MMP-9 antibody (Abcam, ab73734), mouse anti-ASC antibody (Santa Cruz, sc-514414), mouse antiprocaspase1 antibody (Santa Cruz, sc-392736), and mouse antiβactin antibody (Santa Cruz, sc-8432) overnight at 4 °C. Membranes were then washed and incubated with appropriate secondary HRP-conjugated antibody at room temperaturefor 2 hr. Finally, the relative protein expression levels were quanfied byusing the ImageJ software (version 1.8) and normalized to βactin as a control. It should be noted that during Wlottingblotting the IL-1β antibody detected both 33 kDa pro-IL-1β and 17 kDa mature IL-1β, however, only the cleaved mature IL-1β was considered for statistical analysis.

***Determination of caspase-1 enzyme activity***

The enzymatic activity of caspase-1 was determined using a Caspase-1 Colorimetric Assay Kit (BioVision Inc., CAUSA). The assay was based on spectrophotometric detection of the chromophore *p*-nitroanilide (*p*NA) after cleavage from the labeled substrate YVAD-*p*NA at 400 nm. Briefly, total protein was extracted from tissue samples and its concentration was measured according to the Bradford procedure. Then, sampls (100-–200 µg of total protein) were diluted with Lysis Buff (50 µl) and 2× Reaction Buff (50 µl). Samples were then incubated with labeled substrate YVAD-*p*NA at 37 °C 2 hr. Finally, caspase-1 activity was meured byusing a spectrophotometer at 400 nm and expressed as fold change of caspase1 activity in cancerous tissues compared with the activity in the adjacent control tissue.

***Histopathology examination***

TissuesTissue samples were fixed in 10% neutral buffered formalin, dehydrated in graded ethanol, and embedded in paraffin. Sections of 5 µm in thickness were stainetoxylinHematoxyld eosinEin (H& &E) and examined by light microscopy.

***Statistical analysis***

Data analysis was carried out using SPSS software version 16.0 (SPSS Inc., Chicago, IL-USA) and GraphPad Prism software version 6.0 (Graph Pad Software, CA-USA). One-way ANOVA analysis was used for normally distributed variables and Kruskal–Wallis test was performed for abnormally distributed continuous variables. Appropriate multiple comparison tests were performed to determine differences in means between groups (tissues with grade I, tissues with grade III, and normal tissues adjacent to the cancer tissues). Independentsample t-test was used to determine differences in tumor size between grade I and grade III. The correlations between variables were assessed using Pearson’s correlation coefficient. Values are presented as means±SD and significance difference was defied as *P*<0.05. 

## Results

***Demographic characteristics***

As shown in Table 2, demographic characteristics revealed no significant differences in age, weight, height, and BMI between CRC patients at different grades of disease (grade I and grade III). The tumor size was significantly high in grade III compared to grade I (6.1±.05 vs. 3.55±0.6 cm, *P*<0.001). 

***TNM staging of patients***

According to the TNM staging system, 18 patients were classified as stage I and 25 patients were categorized as stage III, as shown in Table 3. Among patients in stage I, 66.6% had tumor invasion to the submucosa (T1), while in the rest of the patients (33.4%) invaded tumors into muscularis propria were observed (T2). In none of the patients from stage I regional lymph nodes were involved (N0) and no distant metastasis was found (M0). In contrast, patients from stage III had tumor invasion into different layers by 16% invasion to the submucosa (T1), 28% to muscularis propria (T2), 24% into subserosa (T3), and 32% tumor invasion into visceral peritoneum (T4). Regional lymph nodes were associateh tumortumors in all of the patients in stage III. In 76% of patients at least one to three (N1) and in 24% more than four (N2) regional lymph nodes were found involved in the disease. However, there was no distant metastasis in these patients (M0).

***Histopathological findings***

**Figure 1:**
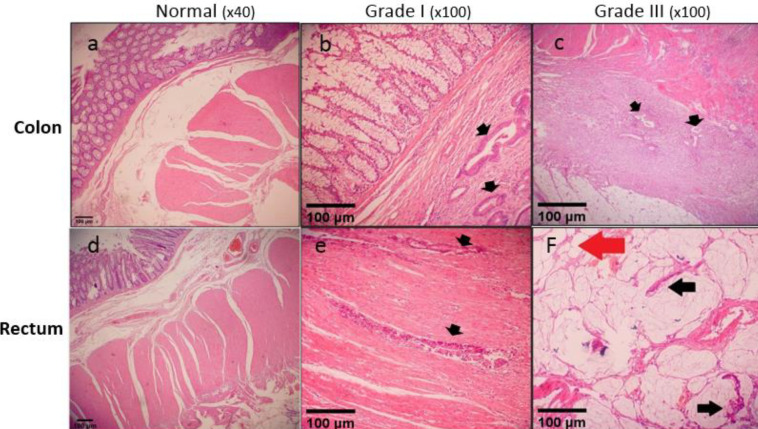
Microscopic assessment of colorectal tissue. (a) Four layers of normal colon consisting of mucosa, submucosa, muscularis propria, and serosa, (b) Malignant cells as glands are visible in the submucosa, (c) Subserosa has been invaded by adenocarcinoma, (d) In lower rectum there is no serosa and perirectal fatty tissue is the deepest layer, (e) Invasion of malignant cells into muscularis propria, and (f) floating malignant cells in mucin pool have reached the perirectal fat (Red arrow). Scale bars in all figures represent 100 µm and the microscopic magnifications are shown as ×40 or ×100. Normal: normal tissue adjacent to the cancer tissue; grade I and III: cancerous colorectal tissue obtained from patients with grade I and III. The black filled arrows point to cancer cells

Tissue samples were fixed in 10% neutral buffered formalin and embedded in paraffin. Sections of 5 µm in thickness were staine (H & E) H&E and examined by light microscopy. Qualitative tumor assessment was performed on histologic sections as gradeI and III Figure 1). Four layers of normal colon consisting of mucosa, submucosa, muscularis propria, and serosa were clearly visible in control tissues (Fiure 1a), whereas malignant cells as glands were found in the submucosa (Figure 1b) and invasion of adenocarcinoma into subserosa was observed (Figure 1c). In the lower rectum, there was no serosa and perirectal fatty tissue is the deepest layer (Figure 1d) but invasion of malignant cells into muscularis propria (Figure 1e) was found. The floating malignant cells in the muciol havehad reached the perirectal fat (Figure 1f). 

***Activation of NLRP3 inflammasome in colorectal cancer***

**Figure 2:**
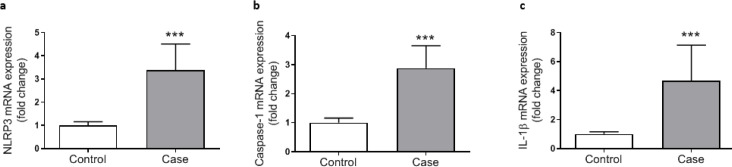
Relative mRNA expression levels of NLRP3 (a), Caspase 1 (b), and IL-1β (c) in control (n=43) and cancerous tissue (n=43) samples, as determined by qRT-PCR. Gene expression levels were normalized to the expression of GAPDH (as housekeeping gene) and fold changes in gene expression were calculated according to the 2^─ΔΔCT^ method. ^“***” ^represents significant difference between groups (*P*<0.001)

**Figure 3:**
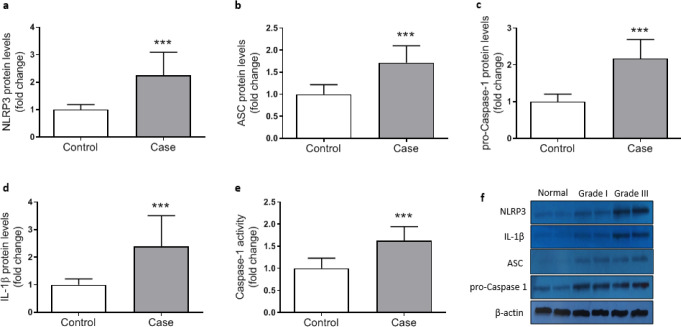
Relative protein levels of NLRP3 (a), ASC (b), pro-caspase-1 (c), and IL-1β (d) and relative caspase-1 activity (e) in control (n=43) and cancerous tissue (n=43) samples, as determined by Western blotting for protein levels (f) and colorimetric assay (for enzyme activity). Protein levels were normalized to β-actin. ^“***”^ represents significant difference between groups (*P*<0.001)

**Figure 4:**
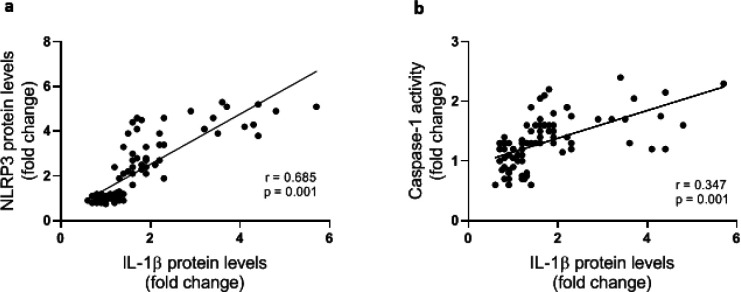
Correlation of NLRP3 protein level (a) and caspase-1 activity (b) with IL-1β protein level in control (n=43) and cancerous tissue (n=43) samples. Protein levels were determined by Western blotting and caspase-1 enzyme activity was assessed by colorimetric assay. The correlation between variables was assessed using Pearson's correlation coefficient

To investigate the involvement of NLRP3 inflammasome activation in colorectal cancer the expression of components of NLRP3 inflammasome complex including NLRat the both mRNA and protein levels), ASC (protein level), caspasat the mRNA and protein levels as well as its enzymatic activity), and ILat the transcriptional and protein levels) were determined. Data analysis showed signiulationup-regulation in the expression of all components of the inflammasome. NLRP3, caspase1, and IL1β gene expressions were significantly higher in cancerous tissue samples compared with the adjacent control tissues (Figure 2). Similarly, markedly increased levels of NLRP3, ASC, procaspase1, and IL1β proteins were detected in colorectal cancer tissues indicating activation of NLRP3 inflammasome (Figure 3). Concurrent to the increase in mRNA and protein levels, significantly higher caspase1 activity was also observed in cancerous tissues compared with the controls (Figure 3e). In addition, based on Pearson’s correlation analysis significant direct correlations were observed between NLRP3 and IL-1β (r=0.685, *P*=0.001) protein levels and between caspase-1 activity and IL-1β protein level (r=.347, *P*=0.001) reconfirming that higher expression of NLRP3 is accompanied with an increase in caspase-1 expression and activity and finally leto the increased production of IL-1β (Figure 4). 

***Correlation of NLRP3 inflammasome activation and progression of CRC***

**Figure 5:**
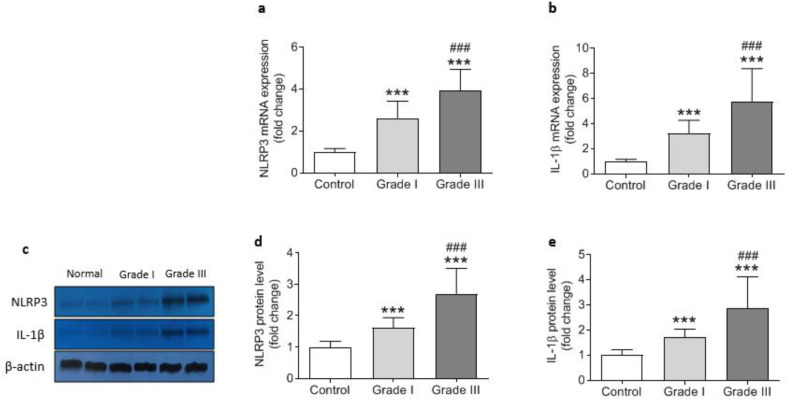
Relative mRNA expression and protein level of NLRP3 and IL-1β in control (n=43) and cancerous tissue samples from grade I (n=18) and grade III (n=25). Gene expression levels (a and b) were determined by qRT-PCR and normalized to GAPDH while the protein levels (c, d, and e) were assessed by Western blotting relative to β-actin. ^ “***” ^represents significant difference compared with control (*P*<0.001) whereas “*###” *indicates difference versus grade I (*P*<0.001)

**Figure 6:**
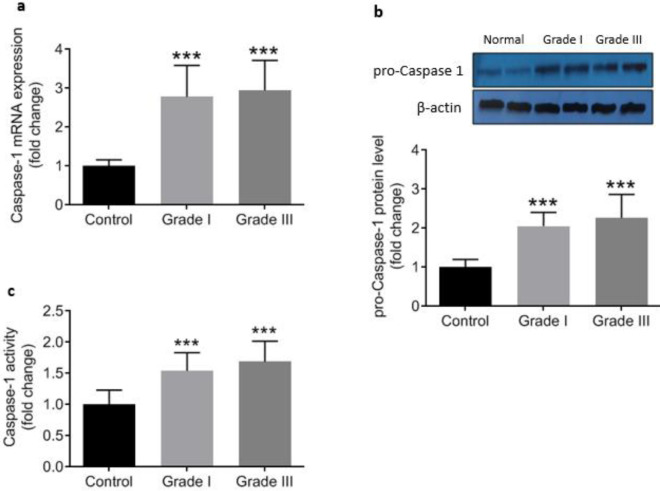
Relative mRNA expression (a), protein level (b), and enzyme activity (c) of Caspase-1, as determined by qRT-PCR, Western blotting, and colorimetric assay, respectively in control (n=43) and cancerous tissue samples from grade I (n=18) and grade III (n=25). ^ “***”^ represents significant difference compared with control (*P*<0.001)

Activation of NLRP3 inflammasome was in correlation with the progression and worsening of the disease. As shown in Fig5, the gene expression and protein levels of NLRP3 were higher in CRC patiet gradeGrade-III than that of patients with grade-I. Likewise, IL1β was found ggulatedup-reguld atthe both mRNA and protein levels in grade-III compared with grade-I (Figure 5). Increasing trends were also detected in gene expression, protein level, and enzyme activity of caspase1 from gradeI to gradeIII in colorectal cancer patients, although it did not reach statistical significance (Figure 6). These data suggest that formation of NLRP3 inflammasome may not be linked to grades of CRC but NLRP3 is independently correlao gardegrade progression, and inflammation can be exacerbated by the progression of cancer. 

***Activation of NLRP3 inflammasome promotes the EMT process***

**Figure 7:**
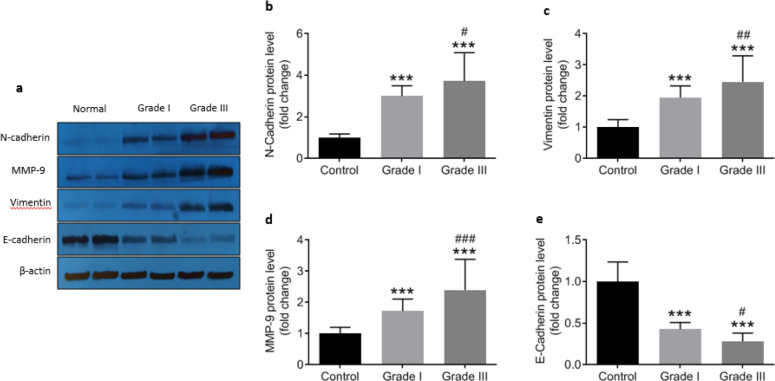
Relative protein levels of N-Cadherin, vimentin, MMP-9, and E-cadherin in control (n=43) and cancerous tissue samples from grade I (n=18) and grade III (n=25).^ “***”^ represents significant difference compared with control (*P*<0.001) whereas ^#^(*P*<0.05), ^##^(*P*<0.01), and ^### ^(*P*<0.001) indicate differences versus grade I

Up-regulation in the expression of NLRP3 inflammasome components (NLRP3, ASC, and caspase1) togetheth the increased production of IL1β were accomith theby promotion of epithelial to mesenchymal transition through enhancement in Ncadherin, vimentin, and MMP9 and reduction in Ecadherin protein levels (Figure 7). As the result of Pearson›s correlation analysis is tabulated in Table 4, NLRP3 was positively correlated with vimentin (r=0.357, *P*=0.019) and MMP9 (r =0.36, *P*=0.018), whereas it was in inverse correlation with E-cadherin (r=0.539, *P*=0.001). Likewise, IL1β as the product of activation of NLRP3 inflammasome showed direct correlation with MMP9 (r = 0.471, *P*=0.001) while it increased by the reduction in Ecadherin (r =0.53, *P*= 0.001). These data clearly showeulationup-regulation of NLRP3 inflammasome components and the consequent increase in the expression of IL-1β are associated with epithelial to mesenchymal transition during colorectal cancer progression *in vivo*.

***TGF*** ***β*** ***expression modulates*** ***the development of CRC***

**Figure 8:**
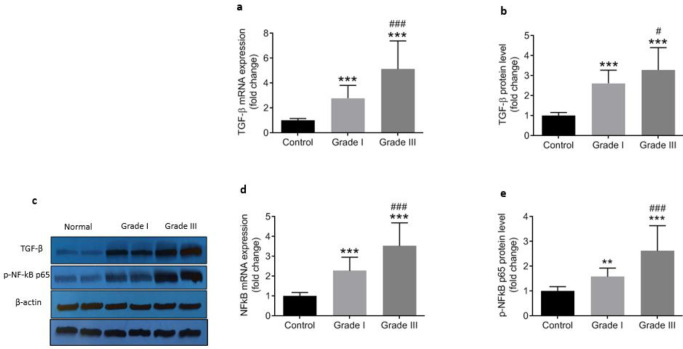
Relative mRNA expression and protein level of TGF-β and NF-κB in control (n=43) and cancerous tissue samples from grade I (n=18) and grade III (n=25). Gene expression levels (a and d) were determined by qRT-PCR and normalized to GAPDH while the protein levels (b, c, and e) were assessed by Western blotting relative to β-actin. ^ ** ^(*P*<0.01) and^ ***^ (*P*<0.001) represent significant difference compared with control whereas ^# ^(*P*<0.05) and^ ###^ (*P*<0.001) indicate differences versus grade I

TGFβ was found remarkably hir atthe both mRNA and protein levels in colorectal cancerous tissue compared with adjacent control tissues, being greater in patients at gradeIII than gradeI (Figure 8). A sulationup-regulation pattern was also observed for NF-κB at the gene expression level and its phosphorylated active protein (p-NF-κB p65) in cancerous tissue samples (Figure 8). The levels of both NF-κB mRNA and its phosphorylated protein were significantly higher in gradeIII than gradeI of disease (Figure 8). Pearson›s correlation analysis showed significantly direct correlation of both TGFβ and NF-κB with markers of EMT including vimentin and Ncadherin and an inverse correlation with Ecadherin (Table 4).

## Discussion

Colorectal cancer (CRC) is a multifactorial disease and the third most common cancer type across the globe (1). It was generally believed that acute inflammation may play a key role in tumor progression, but it is now becoming apparent that initiation and progression of ccers isare associated with chronic inflammation (18). In addition, it is well documented that inflammasomes can play dual roles in cancer progression depending on the specific contexts by providing a promising target in cancer therapy or by acting multifunctionally to induce carcinogenic inflammation, cell growth, and tumor metastasis (19). Initiation and progression of canceition (invohich is, a procby thatwhich epithelial cells lose their polarity and are converted to a more invasive (mesenchymal) phenotype (6). Therefore, it is not surprising that delineation of molecular cross-talk between activation of NLRP3 inflammasomes and EMT-associated markers has become the center of research attention and can be an attractive strategy for reducing the risk of colorectal cancer progression. aim of thitudy wasaimed to determine whether activation of NLRP3 inflammasome is positively or negatively correlated with cancer progression and EMT markers in CRC patients.

Our findings for the first time revealed that NLRP3 inflammasome components such as NLRP3, ASC, and caspaseegulatedup-regulated during CRC progression. This observation confirmed that the NLRP3 inflammasome pathway was activated in colorectal cancer. Similar to our iaoting Deng *et al.* have reported that the cross-talk between colorectal cancer cells and surrounding macrophage cells increased the expression of NLRP3 inflammasome in macrophages, and consequently resulted in the migration and metastasis of colorectal cancer cells (20). Inversely, knockdown of NLRP3 in colorectal carcinoma cells suppressed CRC cell migration *in vitro* and decreased the capacity of invasion (14). 

We also showed that inflammatory cytokine IL-1β, as the end product of NLRP3 inflammasome activation, was er at the both mRNA and protein levels in CRC tissue samples compared with the adjacent control tissues. Interestingly, the activity of caspase1 was remarkably higher in CRC tissue samples compared with control tissues indicating greater production of IL-1β. Since it has previously been reported that upon activation, NLRP3 inflammasome induces proteolytic cleavage and conversion of pro-IL-1β to IL-1β (21) and IL-1β, in turn, promotes proliferation and migration of the lung cancer cells (22) as well as tumor growth and angiogenesis (23). Taking into account all these data, it is therefore proposed that blocking of NLRP3 inflammasome activation may serve as a therapeutic target for preventing lung cancer progression. 

Results of the present study as a first report so far showed that both gene expression and protein levels of NLRP3 were significantly higher in CRC patients at gradeIII than those at gradeI. Likewise, IL-1β was found greater both at the mRNA and protein levels in patients at grade III compared with patients at gradeI. This observation indicated firstly, the presence of a direct correlation between NLRP3 inflammasome activation and production of IL-1β, as its end product, and secondly, strong a correlation between activation of NLRP3 inflammasome (and production of IL-1β) with the worsening of the disease. 

The epithelial to mesenchymal transition (EMT) is a process in which epithelial cells lose their epithelial characteristics including polarity, adherent junctions, and expression of epithelial markers, and gain mesenchymal properties such as motility and invasiveness (6). The present study showed that E-cadherin (as an epithelial marker) is down-regulated whereas concomitantly N-cadherin, vimentin, and MMP-9 (as mesenchymal markers) are up-regulated in colorectal cancer. Intriguingly, the reduction in E-cadherin and increasing of N-cadherin, vimentin, and MMP-9 were found strongly dependent on the severity of disease; being profound at gradeIII compared with gradeI. Our observations are in line with previous reports indicating that the loss of E-cadherin expression is associated with a poor prognosis in gradeIII of colorectal cancer (24), and the overexpression of N-cadherin can provide a clinical prognostic predictor in patients with CRC (25). The increased expression of N-cadherin can also promote both proliferation and migration of colorectal cancer cells by EMT induction (25), as confirmed by the enhancement of the MMP9 level in the present study. We also showed that reduction in the expression of epithelial marker (Ecadherin) had a strong inverse correlation with the production of IL-1β whose association with the progression of the disease has already been reported (22, 23). Together these results suggest that suppression of the EMT process might be a useful candidate therapeutic strategy to retard the progression of CRC; a goal which has been achieved for instan by suppressingof N-cadherin expression using siRNA in lung cancer cell line (26).

Our results also demonstrated that NLRP3 expression was positively correlated with vimentin and MMP9 while a negatively strong correlation was observed between NLRP3 and E-cadherin. In addition, the IL-1β level as the marker of activation of NLRP3 inflammasome had a direct correlation with MMP9 and an inverse relationship with Ecadherin in the present study confirming that activation of NLRP3 inflammasome and initiation of inflammation via production of IL-1β in colorectal cancer is directly associated with the evoking of epithelial to the mesenchymal transition process. These results are agreement with thea previous study indicating that NLRP3 inflammasoation downregulate-regulates the expression of E-cadherin in lung cancer (22). There also several linelines of *in vivo* and *in vitro* evidence confirming that loss of or reduced E-cadherin expression is associated with local lymph node metastasis (27), aggressive phenotypes in colorectal cancer (28), and inasion (29), whereas, metastasis of different cancer types such as colorectal cancer ccompanied with the increased mesenchymal markers including vimentin, N-cadherin, and MMP9 (30). 

The common denominator affecting inflammation and development of cancer is IL-1β level. It has been shown by Jeon M *et al*. (2016) that inhibition of IL-1β expression decreased invasiveness of breast cancer cells through suppression of NFκB activity (31) whereas overexpression of IL-1β in metastatic bladder cancer cells is found responsible for the invasion (32). Moreover, the results of numerous studies in the last wth factor-beta (TGFβ),TGFβ, a potent inducer of cancer-associated EMT (9), plays pivotal roles in the EMT process in colorectal cancer (8). In the present study, we found a significant increase in NFκB and TGFβ expressions concurrent with the progression of the disease, significant negative correlations between NFκB and TGFβ with the epithelial marker of EMT (Ecadherin), and strong positive correlations between NFκB and TGFβ and mesenchymal markers of EMT (vimentin and Mcadherin). These observations support the notion that NFκB and TGFβ play important roles in EMT of colon cancer cells and act through different mechanisms to promote cancer progression and metastasis (33, 34). There is compelling evidence that the role of TGFβ signaling in tumor suppression or development is context- and stage-dependent (35). Moreover, it is postulated that TGFβ/Smad signaling pathway exerts both negative and positive effects on cancer development (36), whereas non-Smad pathways are responsible for the tumor suppression effect of TGFβ. For instance, Smad-dependent cell migration and metastasis can be promoted by mutant p53 (37), and the hyperactive TGFβ/Smad pathway confers poor prognosis in patients with glioma (38). Interestingly, these statements are in line with the concept that TGFβ acts as a tumor suppressor in he early stages of cancer, but paradoxically may act as a tumor promoter in advanced cancers (39). Generally, EMT is affected by TGFβ and WNT signaling pathways and TGFβ pathway is well-established as a strong inducer of EMT in colon cancer cells (14), glioblastoma (40), and breast cancer (34). In accordance with previous findings, our results pointed out that EMT is an important mechanism for invasion and metastasis of colorectal tumor cells, which can be regulated by NFκB and TGFβ.

Taken to account the limitations present in our study, we believe that determination of gelatinase activity of MMP-9, immunohistochemical examination of tissue samples for EMT markers, determination of the other members of NFκB and TGFβ signaling pathways such as Smad, and measurement of other cytokines and adipokines f in cancerous tissue at the gene expres protein levels may strengthstrengthen the results, and conducting of further investigations which entirely overcome all confounder factors are required. 

**Table 1:** List of primers and related sequences used in the study for qRT-PCR

**Genes**	**Forward **	**Reverse **
**TGFβ1**	5’-CTGGCGATACCTCAGCAACC-3’	5’-GTAGTGAACCCGTTGATGTCC-3’
**Caspase-1**	5’-ACAAGTCAAGCCGCACAC-3’	5’-CTCTGTAGTCATGTCCGAAGC-3’
**NLRP3**	5’-CGTCTTTGAGCCTTCTTGGTAG-3’	5’-GTCATTGTCCTGGTGTCTTCC-3’
**IL-1β**	5’-GATGGCTTATTACAGTGGCAATG-3’	5’-TAGTGGTGGTCGGAGATTCG-3’
**NF-κB**	5’-GAAGGTGGATGATTGCTAAG-3’	5’-TGCTGGAGTTCAGGATAAC-3’
**GAPDH**	5’-AAGGCTGTGGGCAAGGTCATC-3’	5’-GCGTCAAAGGTGGAGGAGTGG-3’

GAPDH: Glyceraldehyde 3-phosphate dehydrogenase, IL-1β: interleukin-1β, NF κB: nuclear factor κB, NLRP3: NLR family pyrin domain containing 3, TGFβ: transforming growth factor-β

**Table 2:** Demographic characteristics of the colorectal cancer patients*

**parameters**	**Grade-I (n=18)**	**Grade-III (n=25)**	***P*** **-value**
**Age (year)**	56.36±11.41	58.87±10.87	0.69
**Weight (kg)**	78.65±4.14	76.88±6.24	0.78
**Height (m)**	1.74±0.07	1.75±0.06	0.63
**BMI** ^**^ ** (kg/m** ^2^ **)**	26.05±0.61	25.18±0.41	0.29
**Tumor size (cm)**	3.55±0.76	6.1±1.05	**<0.001**

*Values are presented as mean±SD. Grade-I and Grade-III are patients with colorectal cancer who were classified at grades I and III of disease.

**BMI: body mass index

**Table 3:** Classification of colorectal cancer patients based on the TNM category

**parameters**		**Grade-I (n=18)**	**Grade-III (n=25)**
**Tumor invasion**			
	**T1**	66.6% (n=12)	16% (n=4)
	**T2**	33.4% (n=6)	27% (n=7)
	**T3**	---	24% (n=6)
	**T4**	---	32% (n=8)
**Lymph node involvement**			
	**N0**	100% (n=18)	---
	**N1**	---	76% (n=19)
	**N2**	---	24% (n=6)
**Distant metastasis**			
	**M0**	100% (n=18)	100% (n=25)
	**M1**	---	---

Values are presented as percentages and the number of subjects (n) in each classification

T1: tumor invaded submucosa; T2: tumor invaded muscularis propria; T3: tumor invaded subserosa; T4: tumor invaded visceral peritoneum

N0: no regional lymph node involvement; N1: 1-3 regional lymph node involvement; N2: more than 4 regional lymph node involvement

M0: no distant metastasis; M1: distant metastasis

**Table 4:** Correlation of TGF-β1 with NLRP3, EMT markers, inflammation, and tumor size*

**Variables**	**TGF-** **1**	**N-cad**	**MMP9**	**vimentin**	**E-cad**	**IL-1**	**NLRP3**	**ASC**	**Caspase1**	**NF-** **B**	**Tumor size**
**TGF-** **1**		0.52 **<0.001**	0.268 0.082	0.569 **<0.001**	-0.39 **0.01**	0.19 0.223	0.268 0.082	0.248 0.109	0.22 0.157	0.296 0.054	0.397 **0.008**
**N-cad**			0.223 0.15	0.372 **0.014**	-0.396 **0.009**	0.145 0.355	0.28 0.069	0.087 0.578	0.113 0.469	0.448 **0.003**	0.379 **0.012**
**MMP9**				0.273 0.077	-0.336 **0.028**	0.471 **0.001**	0.36 **0.018**	-0.08 0.607	0.179 0.251	0.205 0.188	0.157 0.315
**Vimentin**					-0.302 **0.049**	0.291 0.058	0.357 **0.019**	0.159 0.31	0.202 0.194	0.446 **0.003**	0.347 **0.023**
**E-cad**						-0.53 **<0.001**	-0.539 **<0.001**	-0.168 0.28	-0.169 0.27	-0.569 **<0.001**	0.508 **0.001**
**IL-1**							0.685 **<0.001**	0.009 0.956	0.347 **0.007**	0.465 **0.002**	0.372 **0.014**
**NLRP3**								0.12 0.443	0.186 0.232	0.572 **<0.001**	0.483 **0.001**
**ASC**									0.124 0.429	0.369 **0.015**	0.223 0.151
**Caspase1**										0.163 0.298	0.163 0.295
**NF-** **B**											0.553 **<0.001**

ASC: Apoptosis-associated speck-like protein containing a CARD, E-cad: E-cadherin, IL-1β: interleukin-1β, N-cad: N-cadherin, MMP9: matrix metalloproteinase-9, NF κB: nuclear factor κB, NLRP3: NLR family pyrin domain containing 3, TGFβ: transforming growth factor-β. *In each cell upper number represents Pearson's correlation coefficient (r) and the lower number shows statistical significance (*P*-value). Negative numbers indicate inverse correlation between two variables

## Conclusion

Ours for the first time so far showed that NLRP3 inflammasome is activated in colorectal cancer and its activation is concurrent with the progression of epithelial to mesenchymal transition and development of CRC. Therefore, NLRP3 inflammasome activation might be responsible for *in vivo* progression of CRC via the EMT process. Although the present study showed that EMT markers are positively correlate, further investigations areinvestigation is required to strongly link the EMT markers to the progression of colorectal c 

## References

[1] Mattiuzzi C, Sanchis-Gomar F, Lippi G (2019). Concise update on colorectal cancer epidemiology. Ann Transl Med.

[2] Chan DS, Lau R, Aune D, Vieira R, Greenwood DC, Kampman E (2011). Red and processed meat and colorectal cancer incidence: meta-analysis of prospective studies. PLoS One.

[3] Chen K, Qiu JL, Zhang Y, Zhao YW (2003). Meta analysis of risk factors for colorectal cancer. World J Gastroenterol.

[4] Binefa G, Rodriguez-Moranta F, Teule A, Medina-Hayas M (2014). Colorectal cancer: from prevention to personalized medicine. World J Gastroenterol.

[5] Armaghany T, Wilson JD, Chu Q, Mills G (2012). Genetic alterations in colorectal cancer. Gastrointest Cancer Res.

[6] Buhrmann C, Yazdi M, Popper B, Kunnumakkara AB, Aggarwal BB, Shakibaei M (2019). Induction of the epithelial-to-mesenchymal transition of human colorectal cancer by human TNF-beta (lymphotoxin) and its reversal by resveratrol. Nutrients.

[7] Tang Y, Shu G, Yuan X, Jing N, Song J (2011). FOXA2 functions as a suppressor of tumor metastasis by inhibition of epithelial-to-mesenchymal transition in human lung cancers. Cell Res.

[8] Zhu QC, Gao RY, Wu W, Qin HL (2013). Epithelial-mesenchymal transition and its role in the pathogenesis of colorectal cancer. Asian Pac J Cancer Prev.

[9] Derynck R, Muthusamy BP, Saeteurn KY (2014). Signaling pathway cooperation in TGF-beta-induced epithelial-mesenchymal transition. Curr Opin Cell Biol.

[10] Glasgow E, Mishra L (2008). Transforming growth factor-beta signaling and ubiquitinators in cancer. Endocr Relat Cancer.

[11] Guo H, Callaway JB, Ting JP (2015). Inflammasomes: mechanism of action, role in disease, and therapeutics. Nat Med.

[12] Song C, He L, Zhang J, Ma H, Yuan X, Hu G (2016). Fluorofenidone attenuates pulmonary inflammation and fibrosis via inhibiting the activation of NALP3 inflammasome and IL-1beta/IL-1R1/MyD88/NF-kappaB pathway. J Cell Mol Med.

[13] Lorenz G, Darisipudi MN, Anders HJ (2014). Canonical and non-canonical effects of the NLRP3 inflammasome in kidney inflammation and fibrosis. Nephrol Dial Transplant.

[14] Wang H, Wang Y, Du Q, Lu P, Fan H, Lu J (2016). Inflammasome-independent NLRP3 is required for epithelial-mesenchymal transition in colon cancer cells. Exp Cell Res.

[15] Wang W, Wang X, Chun J, Vilaysane A, Clark S, French G (2013). Inflammasome-independent NLRP3 augments TGF-beta signaling in kidney epithelium. J Immunol.

[16] Rosen RD, Sapra A (2020). TNM Classification.

[17] Nazari Soltan AS, Rashtchizadeh N, Argani H, Roshangar L, Ghorbani HA, Sanajou D (2018). Dunnione protects against experimental cisplatin-induced nephrotoxicity by modulating NQO1 and NAD(+) levels. Free Radic Res.

[18] Coussens LM, Werb Z (2002). Inflammation and cancer. Nature.

[19] Lin C, Zhang J (2017). Inflammasomes in Inflammation-Induced Cancer. Front Immunol.

[20] Deng Q, Geng Y, Zhao L, Li R, Zhang Z, Li K (2019). NLRP3 inflammasomes in macrophages drive colorectal cancer metastasis to the liver. Cancer Lett.

[21] Kolb R, Liu GH, Janowski AM, Sutterwala FS, Zhang W (2014). Inflammasomes in cancer: a double-edged sword. Protein Cell.

[22] Wang Y, Kong H, Zeng X, Liu W, Wang Z, Yan X (2016). Activation of NLRP3 inflammasome enhances the proliferation and migration of A549 lung cancer cells. Oncol Rep.

[23] Saijo Y, Tanaka M, Miki M, Usui K, Suzuki T, Maemondo M (2002). Proinflammatory cytokine IL-1 beta promotes tumor growth of Lewis lung carcinoma by induction of angiogenic factors: in vivo analysis of tumor-stromal interaction. J Immunol.

[24] Yun JA, Kim SH, Hong HK, Yun SH, Kim HC, Chun HK (2014). Loss of E-Cadherin expression is associated with a poor prognosis in stage III colorectal cancer. Oncology.

[25] Yan X, Yan L, Liu S, Shan Z, Tian Y, Jin Z (2015). N-cadherin, a novel prognostic biomarker, drives malignant progression of colorectal cancer. Mol Med Rep.

[26] Zhang X, Liu G, Kang Y, Dong Z, Qian Q, Ma X (2013). N-cadherin expression is associated with acquisition of EMT phenotype and with enhanced invasion in erlotinib-resistant lung cancer cell lines. PLoS One.

[27] Yan HB, Wang XF, Zhang Q, Tang ZQ, Jiang YH, Fan HZ (2014). Reduced expression of the chromatin remodeling gene ARID1A enhances gastric cancer cell migration and invasion via downregulation of E-cadherin transcription. Carcinogenesis.

[28] Saito T, Masuda N, Miyazaki T, Kanoh K, Suzuki H, Shimura T (2004). Expression of EphA2 and E-cadherin in colorectal cancer: correlation with cancer metastasis. Oncol Rep.

[29] Luo H, Hao X, Ge C, Zhao F, Zhu M, Chen T (2010). TC21 promotes cell motility and metastasis by regulating the expression of E-cadherin and N-cadherin in hepatocellular carcinoma. Int J Oncol.

[30] Hur K, Toiyama Y, Takahashi M, Balaguer F, Nagasaka T, Koike J (2013). MicroRNA-200c modulates epithelial-to-mesenchymal transition (EMT) in human colorectal cancer metastasis. Gut.

[31] Jeon M, Han J, Nam SJ, Lee JE, Kim S (2016). Elevated IL-1beta expression induces invasiveness of triple negative breast cancer cells and is suppressed by zerumbone. Chem Biol Interact.

[32] Matsumoto R, Tsuda M, Yoshida K, Tanino M, Kimura T, Nishihara H (2016). Aldo-keto reductase 1C1 induced by interleukin-1beta mediates the invasive potential and drug resistance of metastatic bladder cancer cells. Sci Rep.

[33] Bates RC, Mercurio AM (2003). Tumor necrosis factor-alpha stimulates the epithelial-to-mesenchymal transition of human colonic organoids. Mol Biol Cell.

[34] Zavadil J, Bottinger EP (2005). TGF-beta and epithelial-to-mesenchymal transitions. Oncogene.

[35] Massague J (2008). TGFbeta in cancer. Cell.

[36] Derynck R, Akhurst RJ, Balmain A (2001). TGF-beta signaling in tumor suppression and cancer progression. Nat Genet.

[37] Adorno M, Cordenonsi M, Montagner M, Dupont S, Wong C, Hann B (2009). A Mutant-p53/Smad complex opposes p63 to empower TGFbeta-induced metastasis. Cell.

[38] Bruna A, Darken RS, Rojo F, Ocana A, Penuelas S, Arias A (2007). High TGFbeta-Smad activity confers poor prognosis in glioma patients and promotes cell proliferation depending on the methylation of the PDGF-B gene. Cancer Cell.

[39] Prud’homme GJ (2007). Pathobiology of transforming growth factor beta in cancer, fibrosis and immunologic disease, and therapeutic considerations. Lab Invest.

[40] Joseph JV, Conroy S, Tomar T, Eggens-Meijer E, Bhat K, Copray S (2014). TGF-beta is an inducer of ZEB1-dependent mesenchymal transdifferentiation in glioblastoma that is associated with tumor invasion. Cell Death Dis.

